# Novel protective role for MAP kinase phosphatase 2 in inflammatory arthritis

**DOI:** 10.1136/rmdopen-2018-000711

**Published:** 2019-01-11

**Authors:** Juliane Schroeder, Kirsty Ross, Kathryn McIntosh, Shilan Jabber, Stuart Woods, Jenny Crowe, Janet Patterson Kane, James Alexander, Catherine Lawrence, Robin Plevin

**Affiliations:** 1 Institute of Infection, Immunity and Inflammation, University of Glasgow, Glasgow, Scotland; 2 Pure and Applied Chemistry, Technology Innovation Centre, University of Strathclyde, Glasgow, Scotland; 3 Strathclyde Institute for Pharmacy and Biomedical Sciences, University of Strathclyde, Glasgow, Scotland; 4 Flagship Biosciences Inc, Massey University, Denver, Colorado, USA

**Keywords:** arthritis, inflammation, chemokines

## Abstract

**Objectives:**

We have previously shown mitogen-activated protein kinase phosphatase 2 (MKP-2) to be a key regulator of proinflammatory cytokines in macrophages. In the study presented here, we investigated the role of MKP-2 in inflammatory arthritis with a particular focus on neutrophils.

**Methods:**

To achieve this, we subjected MKP-2 deficient and wild type mice to collagen antibody induced arthritis, an innate model of arthritis, and determined disease pathology. To further our investigation, we depleted neutrophils in a prophylactic and therapeutic fashion. Last, we used chemotaxis assays to analyse the impact of MKP-2 deletion on neutrophil migration.

**Results:**

MKP-2^-/-^ mice showed a significant increase in disease pathology linked to elevated levels of proarthritic cytokines and chemokines TNF-α, IL-6 and MCP-1 in comparison to wild type controls. This phenotype is prevented or abolished after administration of neutrophil depleting antibody prior or after onset of disease, respectively. While MCP-1 levels were not affected, neutrophil depletion diminished TNF-α and reduced IL-6, thus linking these cytokines to neutrophils. In vivo imaging showed that MKP-2^-/-^ mice had an increased influx of neutrophils into affected joints, which was higher and potentially prolonged than in wild type animals. Furthermore, using chemotaxis assays we revealed that MKP-2 deficient neutrophils migrate faster towards a Leukotriene B4 gradient. This process correlated with a reduced phosphorylation of ERK in MKP-2^-/-^ neutrophils.

**Conclusions:**

This is the first study to show a protective role for MKP-2 in inflammatory arthritis.

**Key messages**

**What is already known about this subject?**

- Previously, we have demonstrated a role for mitogen-activated protein kinase phosphatase 2 (MKP-2) in regulating macrophage function in parasitic infections.

**What does this study add?**

- Here, we demonstrate a novel role for MKP-2 in regulating inflammatory arthritis through the regulation of neutrophil function.
- This study therefore further highlights a prominent role for MKP-2 in regulating immune cell function and pathological outcomes.

**How might this impact on clinical practice?**

- Patients presenting with arthritis could be screened for MKP-2 expression.
- Gene therapy could be used as a tool to rectify decreased or absent MKP-2 expression in patients.

## Introduction

Rheumatoid arthritis (RA) is a disabling condition affecting approximately 0.5%–1% of the population in Europe and North America,^1^ characterised by persistent pain and stiffness of the joints, progressive joint destruction, functional disability and premature mortality. As a multifactorial disease, RA involves both the innate and the adaptive arms of the immune system. Numerous cellular and clinical studies have implicated the involvement of multiple cell types including T cells, B cells, macrophages, neutrophils depending on the immune context; yet, despite intensive study, there are still key deficits in understanding the principal mechanisms on how inflammatory arthritis develops.

A major signalling pathway implicated in the pathogenesis of RA is the MAP kinase pathway involving MAP kinases ERK, p38 and JNK.^2 3^ In RA, all three kinases are activated and implicated to different extents in joint inflammation, pannus formation and joint destruction. Of these three kinases, ERK has recently gained more interest, with one study demonstrating the importance of ERK in synovial cell death, where it was shown to regulate TNF-α-induced expression of B-cell activating factor through an increase in hypoxia-inducible factor-1α.^4^ Another study highlighted a role for ERK in TNF-α-induced IL-8 expression in synovial fibroblasts, which contributes to synovitis.^5^

The kinetics and magnitude of MAP kinase activities is closely regulated by a family of dual specific phosphatases (DUSPs) known as the MAP kinase phosphatases.^6^ Three major classes exist, each with overlapping subcellular expression, kinase specificity and regulation by cellular stimulation.^7^ In contrast to studies involving the MAP kinases, there are very little data investigating the role of DUSPs in regulating RA. However, it has been shown that the deletion of mitogen-activated protein kinase phosphatase 1 (MKP-1) leads to enhanced severity of collagen induced arthritis (CIA) and increased cytokine release,^8^ while DUSP2 (PAC1) deficient mice showed a reduced response in the KxB/N model of inflammatory arthritis.^9^

MKP-2 (DUSP4) is a type 1 nuclear phosphatase^10^ specific for ERK and JNK in vitro. As a type 1 DUSP, MKP-2 has been viewed as a surrogate to the prototypic MKP-1 and less extensively examined.^11^ However, more recent studies have identified distinct roles for MKP-2 in different cellular processes and associated pathologies such as tumour development,^12 13^ cancer resistance,^14^ cardiac myopathy,^15^ infection^16^ and inflammation. Recent studies using KO models have shown that MKP-2 protects against septic shock^17^ and plays a role in regulating T cell function.^18^ Moreover, MKP-2 deletion mice developed in our laboratory have been shown to be highly susceptible to parasite infection with *Leishmania mexicana* and *Toxoplasma gondii* due to alterations in macrophage iNOS/arginase expression.^19 20^ Bone marrow-derived macrophages that have been stimulated with lipopolysaccharide (LPS) showed an increase in proinflammatory IL-6, IL-12, TNFα and a decrease in anti-inflammatory IL-10 in the absence of MKP-2^19^. This cellular and functional phenotype marks MKP-2 as distinct from other MKPs and therefore this DUSP has the potential to play a unique role in the pathogenesis of other diseases.

In this study, we examined the potential role of MKP-2 in the pathogenesis of RA using MKP-2 deficient mice and the collagen antibody-induced arthritis (CAIA) model with a specific focus on neutrophil function and migration.

## Materials and methods

### Mice

Male MKP-2 wild-type and deficient mice were bred onto a C57BL/6 background as previously described^19^ and maintained under specific pathogen-free conditions in the animal facilities at the University of Strathclyde. Animals were used at 6–9 weeks of age and were age matched within each experiment. All experiments were conducted under Project Licenses, RP (PPL60/3439), JA (PPL60/3929) and CEL (PPL60/4297) granted by the UK Home Office and approval from the local ethical committee.

### Collagen antibody-induced arthritis and neutrophil depletion

Mice received a single dose (2 mg) of ArthritoMab (MD Bioscience) i.p. on day 0, followed by a single dose (100 µg i.p.) of *E. coli* O55:b5 LPS (MD Bioscience) i.p. on day 3 (n=5). Negative control mice received either phosphate buffered saline (PBS) on day 0 and LPS on day 3 (n=3) or PBS on both days (n=2) again i.p. Body weight, paw measurements and disease score were monitored daily. For paw measurements, both front and rear paw thickness were determined with a calliper and the measurements from day 0 were deducted to obtain the inflammation induced increase in paw size.

The arthritic disease score was determined as previously described:^21^ 0: no reaction, normal; 1: mild but definite redness and swelling of the ankle or apparent redness and swelling limited to individual digits, regardless of the number of digits; 2: moderate redness and swelling of the ankle; 3: severe redness and swelling of the entire paw including digits; 4: maximally inflamed limb with involvement of multiple joints. The score was averaged for the front paws and rear paws for each mouse.

Neutrophils were depleted in vivo with a single dose of 0.25 mg NIMP-R14 (AdipoGen) i.p. as previously described^22^ and depletion monitored using flow cytometry. To control for NIMP-R14 neutrophil depletion, we used an isotype control: LEAF^TM^ Purified Rat IgG2b,κ Isotype control Antibody (BioLegend, UK).

### Histology

After termination of the experiment, the front and rear paw and knee of each mouse were removed, skinned and fixed in 10% formalin (Sigma-Aldrich). Samples were decalcified in 5.5% EDTA for 30 days, washed and stored in 70% ethanol at 4°C. For processing, samples were paraffin-embedded under vacuum and sections stained with H&E at the University of Glasgow Veterinary School, before being scored blindly by a pathologist as previously described.^23^

### Flow cytometry

Seventy microlitres of blood were taken with heparinised glass capillaries from the tail. After blocking unspecific binding with 10% mouse serum and Fc receptor blocking antibodies (anti-mouse CD16/32, eBioscience, UK), the cell surface was stained with conjugated antibodies for CD11b (Pe-Cy7; eBioscience, UK), and Ly-6G (IA8 clone, APC-Cy7; BioLegend) for 45 min at 4°C. Red blood cells were lysed using FACS Lyse (BD Bioscience) for 10 min after which cells were washed and resuspended in 200 µl PBS. Cells were analysed on the FACS Canto flow cytometer (BD Bioscience) using FACS Diva software.

### ELISA

Mouse serum and joint homogenates were analysed individually for the presence of IL-6, TNF-α, IL-1β and MCP-1 using the ELISA MAX Deluxe Set (BioLegend) following manufacturer’s instructions. Absorbance was read at 450 nm using a plate reader (Spectramax 190, Molecular Devices, USA) and concentrations were determined against standard curves.

### In vivo imaging (IVIS)

Mice received 200 mg/kg luminol i.p. and were anaesthetised for imaging after 10 min.^24^ Images were taken using the IVIS Spectrum (Caliper Life Sciences) with medium binning and 5 min exposure time after which mice were allowed to recover. Images were analysed using the Living Image software (Caliper Life Sciences). Briefly, regions of analysis of the same size were drawn using the photographs and adjusted as required using the overlay. Values were averaged from the two front and rear paws, respectively, and for each individual mouse and the background deducted. Values are expressed as photons per second (p/s).

### Chemotaxis assays

Bone marrow neutrophils from MKP-2^+/+^ and MKP-2^-/-^ mice were isolated on LS magnetic columns using the Ly-6G positive selection kit (Miltenyi Biotec GmbH, Germany) following manufacturer’s instructions. Chemotaxis was assessed using 24-well transwell plates containing polycarbonate filter membranes of 10 µm thickness and 3 µm pore size (Corning). Neutrophils were resuspended in complete Roswell Park Memorial Institute (RPMI) media at 2×10^5^/100 µl and were added to the upper chamber. The lower chamber was filled with 600 µl complete RPMI supplemented with either nothing (negative control), LPS (0.5 µg/mL), C5a (20 nM) or LTB_4_ (300 nM). Each variable was carried out in triplicate. Following 3 hour incubation at 37°C, the contents of the lower chamber were collected and counted. Results were expressed as percentage migration ((number of migrated cells/total number of cells) ×100).

### Western blotting

Neutrophils were isolated as described above and re-suspended in complete RPMI, 1×10^6^ cells were used per treatment point. Following stimulation samples were analysed by Western blotting as outlined previously.^19^

### Statistics

Statistical analysis was performed using GraphPad Prism (V.4.0, San Diego, California, USA). All error bars are shown as SE of the mean (SEM), with two-tailed unpaired t-test, with the exception of Western blots, which were one-way ANOVA with Bonferroni’s post-test (*p<0.05, **p<0.01, ***p<0.001).

## Results

### MKP-2 deficiency results in increased inflammation and disease pathology

**Figure 1:**
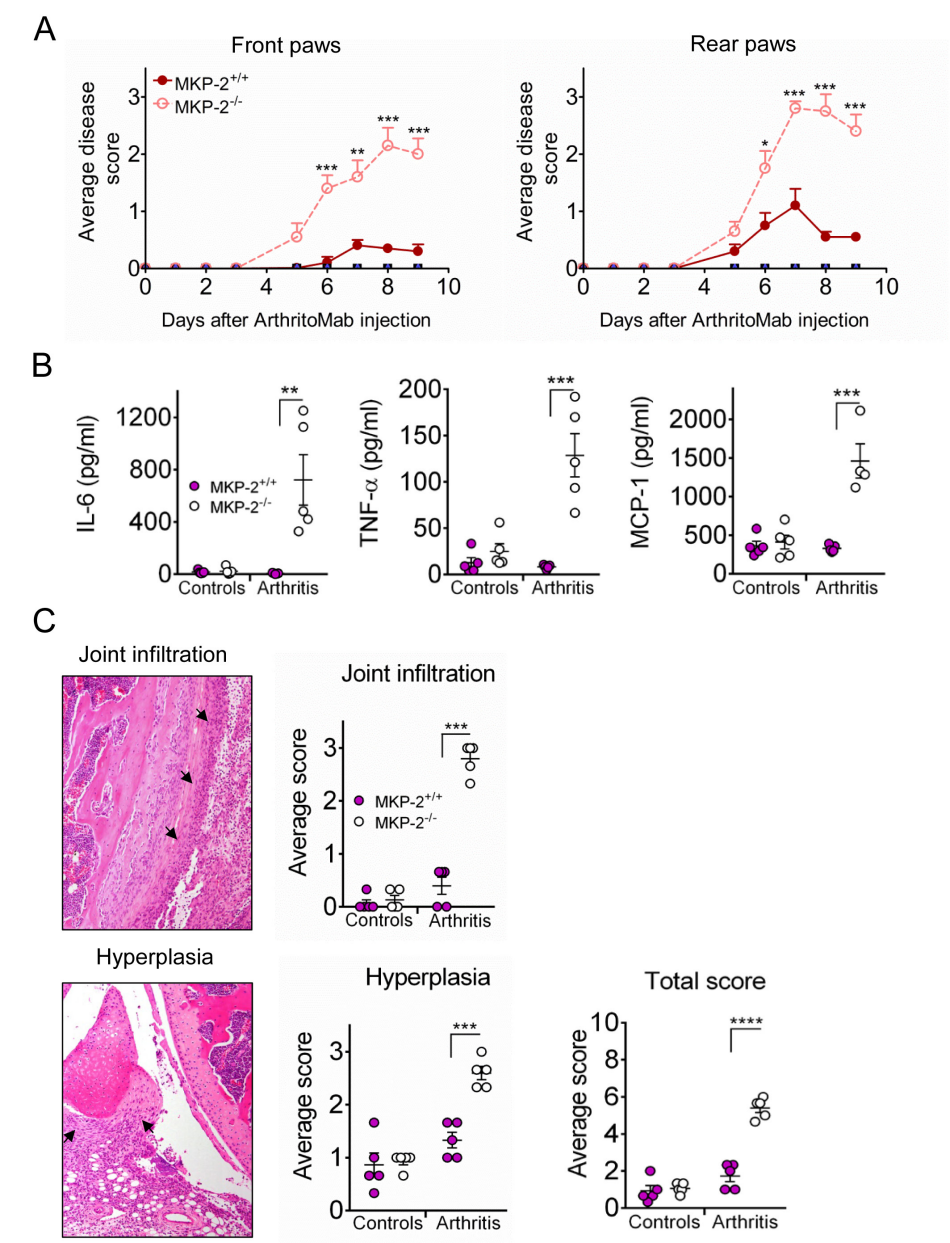
MKP-2 deficiency results in increased inflammation and arthritis related pathology. Mice received a single dose of ArthritoMab at day 0 and a single injection of LPS at day 3. Negative control groups received either PBS on both days or PBS on day 0 followed by a single dose of LPS at day 3. Disease development was observed over a period of 9 days. (A) Arthritic score was determined as described in materials and methods, MKP-2^+/+^ (solid line and symbols), MKP-2^-/-^ (dashed line and open symbols). (B) Serum cytokines were determined by ELISA, controls are a combination of all negative control groups. (C) H&E stained tissue sections (left panels) of front paw, rear paw and knee joints were scored blindly and evaluated for infiltration and hyperplasia (right panels). Arrows point to the respective pathology shown on a representative example of an MKP-2^-/-^ knee joint. Scores of all three joints were averaged per mouse and plotted as MKP-2^+/+^ (solid bars) and MKP-2^-/-^ (open bars). For the total score, scores for joint infiltration and hyperplasia were summed for each individual mouse. All error bars are shown as SE of the mean (SEM). *P≤0.05, **P≤0.01, ***P≤0.001, ****P<0.0001, two-tailed unpaired T test. Data shown are representative of three independent experiments (mouse numbers; pilot—PBS ctrl=2, LPS ctrl=2, ArthritoMab/LPS=6; Exp 2—PBS ctrl=4, LPS ctrl=6, ArthritoMab/LPS=10; Exp 3—PBS ctrl=4, LPS ctrl=6, ArthritoMab/LPS=10 *(Totals across MKP^+/+^ and MKP^-/-^ genotypes, with equal numbers in each).

MKP-2 deficient mice showed a significant increased disease score compared with wild type mice for both front and rear paws. In contrast, control mice showed no signs of pathology (figure 1A). Consistent with an increased disease score, these mice showed a significant increase in paw size (online supplementary figure S1). Analysis of the serum at day 9 showed a dramatic increase of cytokines IL-6 and TNF-α as well as chemokine MCP-1 in MKP-2^-/-^ mice compared with wild type and control mice (figure 1B). We further determined the presence of these cytokines in joint homogenates. However, while slightly increased in MKP-2^-/-^, these results are not statistically significant (online supplementary figure S2).

10.1136/rmdopen-2018-000711.supp1 Supplementary data

10.1136/rmdopen-2018-000711.supp2 Supplementary data

Histology on H&E stained sections of front paws, rear paws and knees showed a significant joint pathology in MKP-2^-/-^ mice with increased infiltration and hyperplasia (figure 1C). Taken together, these results indicate that MKP-2 deficiency results in significant disease pathology in collagen antibody-induced arthritis.

### Inflammation is linked to increased neutrophil migration into the joints and can be influenced by neutrophil depletion

**Figure 2:**
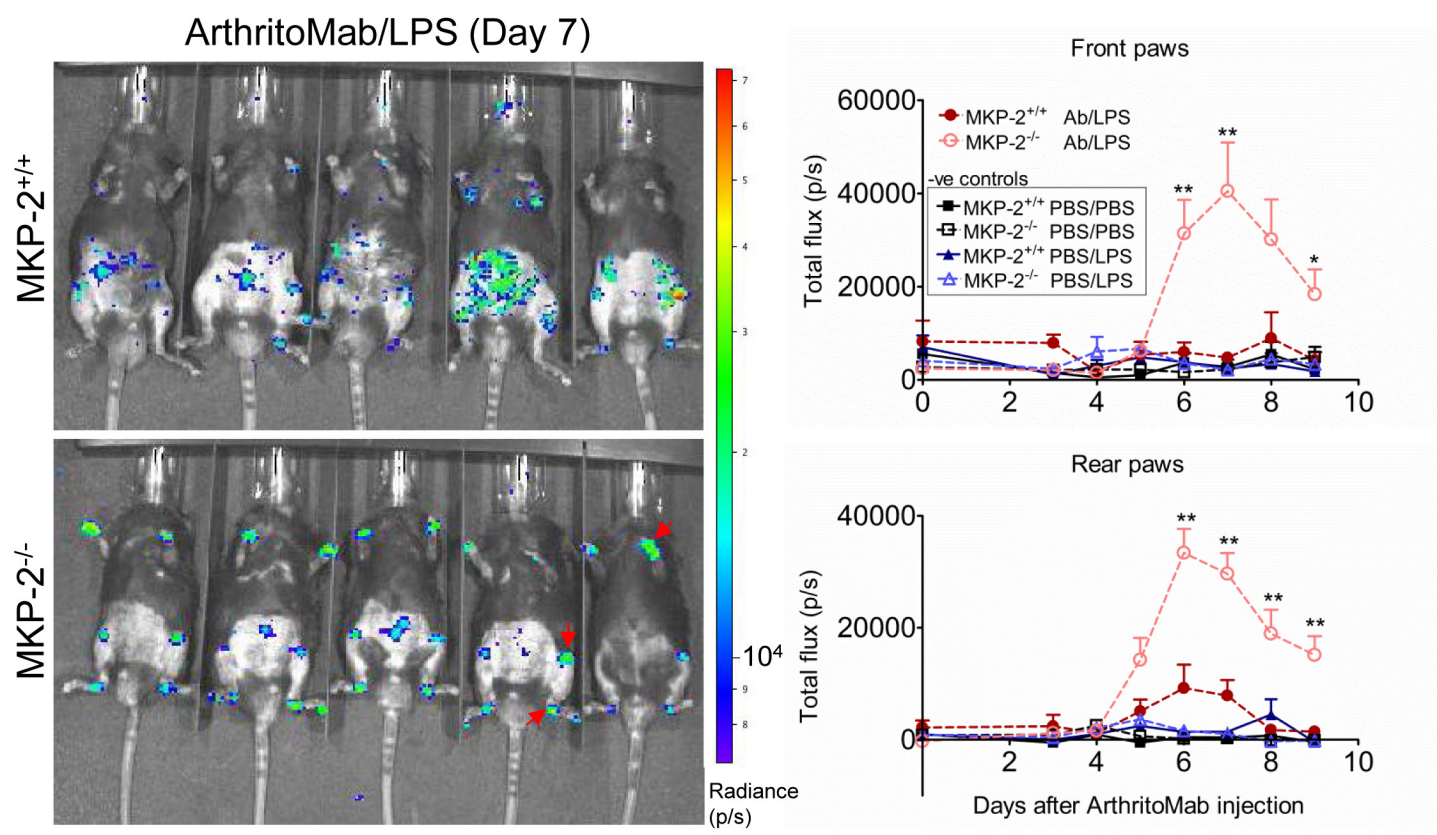
MKP-2^-/-^ mice show an increased neutrophil infiltration into the joints. Mice received a single dose of ArthritoMab (AB) at day 0 and a single injection of LPS at day 3. Negative control groups received either PBS on both days or PBS on day 0 followed by a single dose of LPS at day 3. Disease development was observed over a period of 9 days. Neutrophils were visualised 10 min after i.p. injection of luminol and resulting bioluminescence was detected using in vivo imaging. Shown is a representative image from day 7 (left panels) and the quantification over the duration of the experiment for front and rear paws (right). Red arrows indicate neutrophil influx. Values are expressed as photons per second (P/S). All error bars are shown as SE of the mean (SEM). *P≤0.05, **P≤0.01, two-tailed unpaired T test. Data shown are representative of three independent experiments (mouse numbers; pilot—PBS ctrl=2, LPS ctrl=2, ArthritoMab/LPS=6; Exp 2—PBS ctrl=4, LPS ctrl=6, ArthritoMab/LPS=10; Exp 3—PBS ctrl=4, LPS ctrl=6, ArthritoMab/LPS=10 *(Totals across MKP^+/+^ and MKP^-/-^ genotypes, with equal numbers in each).

Neutrophils have been previously linked to arthritic disease.^25^ In order to visualise myeloperoxidase-containing neutrophil influx into the joints, mice were injected with luminol. In vivo imaging revealed that MKP-2 deficient mice showed a significant increase in neutrophils in the joints compared with wild type and control mice (figure 2).

**Figure 3:**
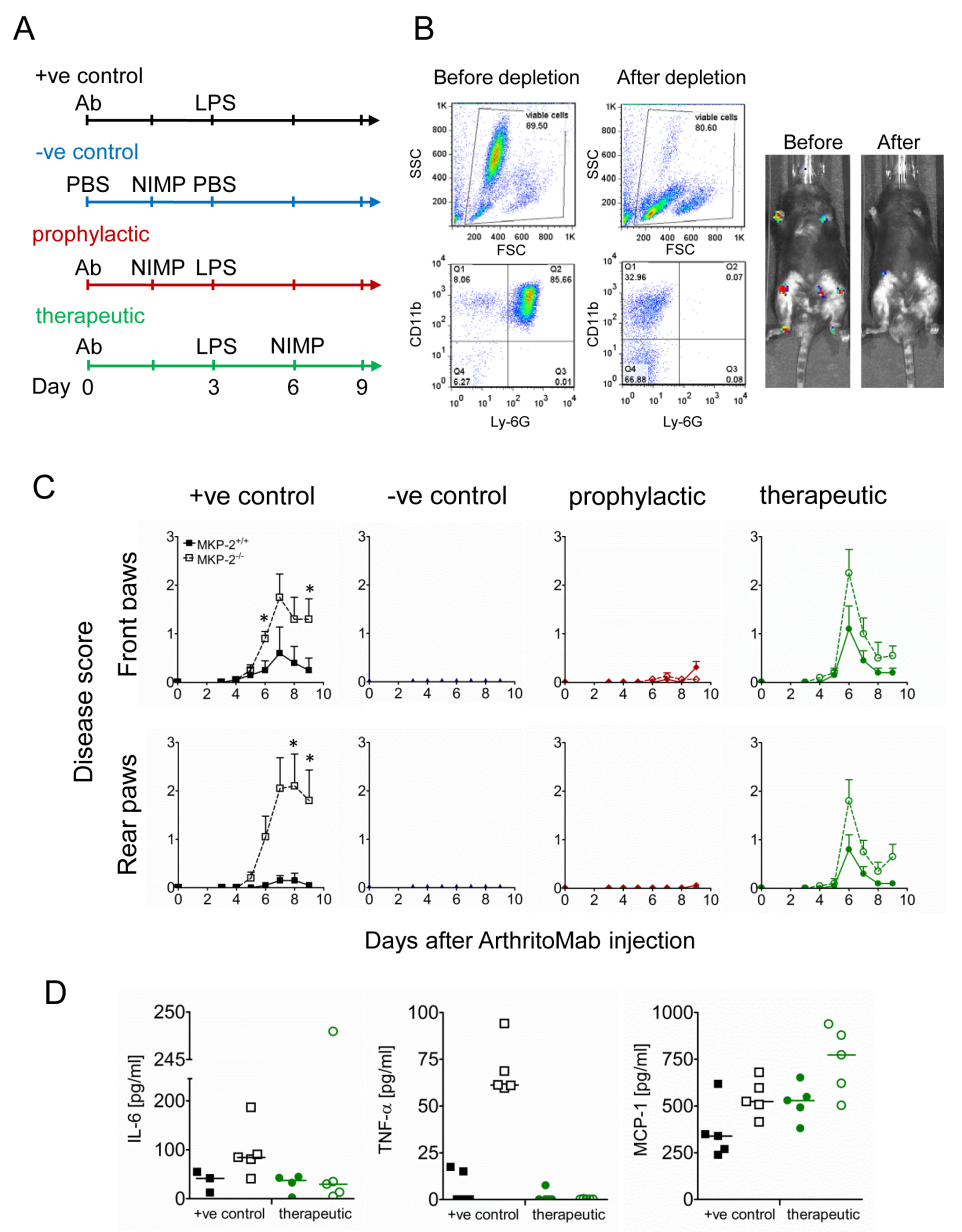
Neutrophil depletion prevents and ameliorates inflammation. Mice were divided into four groups of five animals per genotype and received treatment as shown in (A). All groups but the negative controls received ArthritoMab at day 0 and LPS at day 3. Neutrophils at day 3 were depleted 6 hour prior to i.p. injection of LPS or PBS in the prophylactic and negative control groups, respectively. The therapeutic groups received NIMP after onset of disease at day 6. (B) Neutrophil depletion from blood before and after treatment with NIMP from MKP-2^-/-^ mice from the therapeutic group was monitored using flow cytometry with specific staining for CD11b and Ly-6G (left panels) and also via bioluminescence (right panels) following luminol injection. (C) Disease scores were determined as described in materials and methods, closed symbols depict MKP-2^+/+^ mice and open symbol depicts MKP^-/-^ mice. (D) Serum cytokines were determined using ELISA. All error bars are shown as SE of the mean (SEM). Data shown are representative of two independent experiments. (Mouse numbers; Exp 1: ArthritoMab/LPS=10, PBS/NIMP=10, Prophylactic=10 and Therapeutic=10; Exp 2: ArthritoMab/LPS=6, PBS/NIMP=0, Prophylactic=0 and Therapeutic=6; *(Totals across MKP^+/+^ and MKP^-/-^ genotypes, with equal numbers in each).

We next investigated how depletion of neutrophils affects disease development. Mice were divided into four groups; an overview of the respective treatments is shown in figure 3A. Mice in the positive control group (+ve control) received ArthritoMab at day 0, followed by LPS at day 3 and in addition received an isotype control instead of the neutrophil depletion antibody. These mice developed pathology with MKP-2^-/-^ showing higher disease scores similar to previous observations. Mice of the negative control group (–ve control) were injected with PBS on both days but in addition received neutrophil depleting antibody NIMP-R14 6 hour prior to the second PBS injection to ensure that neutrophil depletion on its own does not influence disease.

The successful depletion of neutrophils was monitored by flow cytometry and by in vivo imaging (figure 3B) and as expected, these mice did not show any significant pathology. Mice of the prophylactic group were treated with NIMP-R14 6 hours prior to LPS injection and before the onset of disease. No disease pathology in respect to arthritis was found in these mice. Similar effects have been found in the therapeutic group, where neutrophil depletion at day 6, after onset of disease, resulted in rapid amelioration of disease symptoms (figure 3C).

Concentrations of the cytokines TNF-α and IL-6 were below detection (TNF-α) or down to levels of the negative controls (figure 3D and online supplementary figure 4). Interestingly, concentrations of MCP-1 were unaffected by neutrophil depletion, thus suggesting that neutrophils are not the source of this chemokine.

10.1136/rmdopen-2018-000711.supp4 Supplementary data

At this point, most animals in the prophylactic group treated with the NIMP-R14 antibody prior to LPS became severely ill and showed signs of endotoxic shock. Although mice in this group made a full recovery, the serum across the groups showed a high variation of IL-6, MCP-1 and TNF-α (online supplementary figure S4); therefore, no conclusion can be drawn as these are more likely to be the result of a cytokine storm than arthritis. For this reason also, this experiment was only conducted twice, and for the second experiment, the prophylactic group was omitted due to the previous endotoxic shock observed.

Histological analysis of affected joints showed that treatment with neutrophil depleting antibody abolished arthritis related pathology in MKP-2^-/-^ mice (online supplementary figure S3) to the level of naive mice. Overall, these experiments point to neutrophils as the main mediator of inflammation since specific depletion of this cell type prevents or ameliorates disease and cytokine levels are ablated after neutrophil depletion.

10.1136/rmdopen-2018-000711.supp3 Supplementary data

### MKP-2 deficient neutrophils show increased chemotaxis

**Figure 4:**
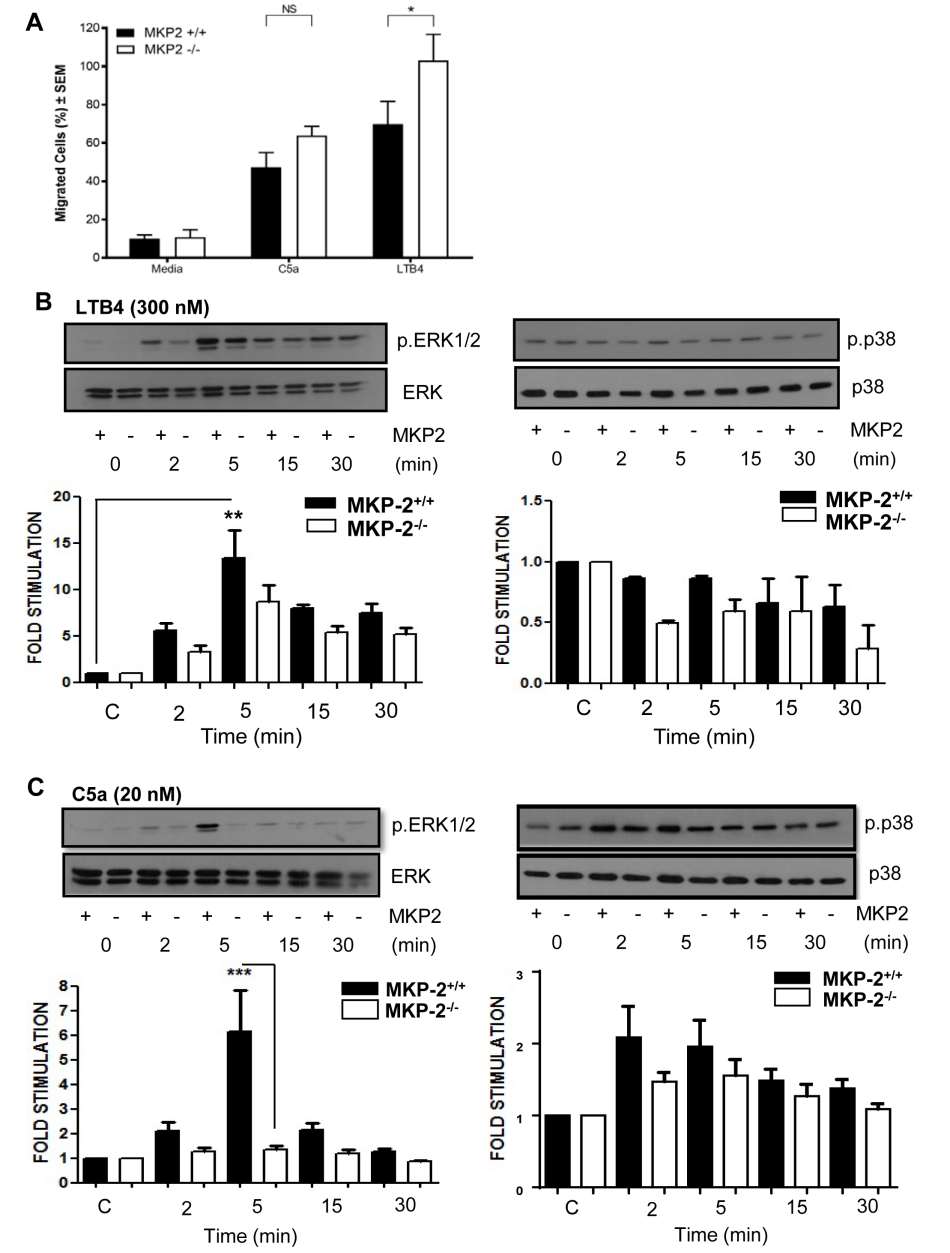
MKP-2 deficient neutrophils show increased chemotactic activity in vitro. (A) Purified neutrophils were subjected to Transwell migration assays using LPS (0.5 µg/mL), C5a (20 nM) and LTB_4_ (300 nM) as chemoattractants. Migrating cells are shown as percentage of the overall population. (B) Neutrophils were stimulated with LTB_4_ (300 nM) over a time course and cell extracts were analysed for the presence of phosphorylated ERK and p38. (C) Neutrophils were stimulated with C5a (20 nM) over a time course and cells’ extracts analysed for the presence of phosphorylated ERK and p38. Membranes were stripped and reprobed for their respective totals. All error bars are shown as SE of the mean (SEM), *p≤0.05, **p≤0.01, ***p≤0.001. (A) Two-tailed unpaired t test, (B,C) One-way ANOVA with Bonferroni’s post-test. (C,D) Closed symbols depict MKP-2^+/+^ mice and open symbol depict MKP^-/-^ mice. Data shown are representative of three independent experiments (mouse numbers—four mice from each genotype, so a total of eight were used per each independent experiment).

We next performed transwell assays to determine if the increased migration is a result of MKP-2 deficiency. As chemo-attractants we chose C5a and Leukotriene B4 (LTB_4_) as these agents are known to have strong chemotactic effects on neutrophils. Neutrophils showed an increased migration towards both C5a and LTB_4_. The migratory effect was significantly enhanced in MKP-2^-/-^ neutrophils in response to LTB_4_ (p<0.05), but no significant difference was seen with C5a (figure 4A).

We then sought to correlate this difference with changes in MAP kinase activity. We found that LTB_4_ strongly phosphorylated ERK in both groups; however, there was no significant difference between MKP-2^-/-^ and MKP-2^+/+^ neutrophils (figure 4B). LTB_4_ did not appear to activate the p38 pathway and therefore no comparisons could be made. In comparison we found that in MKP-2^+/+^ neutrophils, C5a gave a strong activation of ERK peaking at 5 min and this response was markedly reduced in MKP-2^-/-^ neutrophils (p<0.001) (figure 4C). C5a was also able to phosphorylate p38 MAPK, although to a smaller level (n.s), and this was not significantly altered by DUSP4 deletion (figure 4C). In contrast, no JNK phosphorylation was detected in any of the samples (not shown).

## Discussion

Using MKP-2 deficient mice developed in our laboratory, we present for the first time evidence that MKP-2 plays a key protective role in inflammatory arthritis. MKP-2 has been implicated in both inflammatory responses and infection with particular emphasis on macrophages and T-cell function. In this study, we show that in an innate model of arthritis neutrophils are also influenced by MKP-2, and MKP-2 deficient neutrophils migrate to the joint more rapidly to mediate join destruction through enhanced cytokine release.

In this study, enhanced damage to the joint in MKP-2^-/-^ mice was accompanied by a marked increase in serum levels of MCP-1, IL-6 and TNFα. This is in line with observations made in MKP-1^-/-^ mice which received LPS injections^8^ and suggests common regulatory functions between nuclear inducible DUSPs. However, these results are different from another DUSP-4 deletion model which showed reduced serum levels of TNFα in response to LPS.^17^ Differences in the dose of LPS and the time at which TNFα was measured is likely to explain the differences; however, in our hands, MKP-2^-/-^ macrophages also show enhanced TNFα production,^19^ suggesting an intrinsic difference in cytokine production in DUSP-4 deficient cells of myeloid origin compared with WT.

Nevertheless, to our surprise, depletion of neutrophils completely abrogated serum TNFα, which indicates that neutrophils are the main producer of this cytokine in the CAIA model. This is consistent with other studies indicating the potential of neutrophils to be a source of TNFα and other related ligands following innate immune challenge.^26^ Interestingly, MCP-1, which is associated with inflammatory arthritis, was not affected suggesting a different source. While many cells are able to produce MCP-1, it has been shown that monocyte/macrophages are the main producer.^27^ When examining joint pathology, we found that neutrophil depleted mice showed a significantly reduced pathology score. This raises the question as to what extent each cytokine/chemokine contributes to joint destruction. It is known that proinflammatory cytokines, TNFα, IL-6 and IL-1 can indirectly trigger osteoclastogenesis by upregulating RANKL on the surface of osteoblasts.^28^ On the other hand, MCP-1 induces RANKL expression on osteoclast precursor cells, a process which is ERK dependent.^29^ It is therefore possible that the lack of TNFα caused after neutrophil depletion results in a decrease of RANKL on osteoblasts, crucial for osteoclast activation, which would explain why the presence of MCP-1 alone did not result in joint damage. This would be something that could be investigated in future studies.

Since neutrophils appear to be the key cell type responsible for the pathology observed, we addressed the question whether MKP-2 deficiency alters chemotactic activity. Isolated neutrophils displayed migration towards both C5a and LTB_4_; however, MKP-2^-/-^ neutrophils showed a significantly increased migration towards LTB_4_, a potent chemoattractant to neutrophils, and indeed Finsterbusch and coworkers have shown that neutrophils recruited by these chemoattractants release TNFα leading to microvascular leakage and inflammation.^30^ Recent studies have revealed that LTB_4_ is a key mediator in a complement, lipid, cytokine and chemokine cascade that first initiates and then sustains neutrophilic inflammation in inflammatory arthritis^31^

Stimulating neutrophils with either LTB_4_ or C5a resulted in a strong phosphorylation of ERK which was reduced following MKP-2 deletion. Work by Liu *et al* showed that ERK is a negative regulator of migration^32^ and this would be consistent with our finding that in MKP-2^-/-^ neutrophils ERK is reduced, negative regulation is abrogated and migration enhanced. In contrast, p38 MAPK, believed to provide a positive chemotaxis signal, was not significantly altered in our studies. However, it should be noted that our expectation from a signalling perspective is that ERK activation may have been enhanced due to lack of regulatory dephosphorylation by MKP-2. This suggests MKP-2 deletion may have effects which regulate other components of the ERK cascade including receptor expression and functional coupling to upstream intermediates. A number of studies including our own have also suggested that MKP-2 can regulate cellular function in a way which is not dependent on the normal predicted effects on ERK and JNK.^11 33 34^ Indeed, recently MKP-2 has been shown to interact and regulate the cell cycle protein VRK-1 independently of phosphatase activity suggesting multiple cellular effects.^35^ Another coincidental explanation may be pathway redundancy and/or cross talk with MKP-1, such that ERK signalling is altered in a different way.^17^

Finally, another possibility, not examined in this study, is that MKP-2 may regulate apoptosis and indeed MKP-2 deficient neutrophils are present in far greater numbers and appear to reside in the joint for longer, possibly prolonging the release of cytokines. This would be consistent with studies that show delayed neutrophil apoptosis in response to LTB_4_ 36 and *L. major* infection.^37^ Indeed, MKP-2 deletion results in the inhibition of B- cell apoptosis^38^ and also enhances T cell proliferation^18^ consistent with this idea.

Overall, our novel findings suggest that MKP-2 has the potential to play a major role in regulating RA due to a direct effect on neutrophil function, with the potential to effect macrophages, osteoclast formation and bone development. The use of specific conditional knockout mice would allow further dissection of these events. However, it remains that MKP-2 may be an attractive target for gene replacement therapy.
