# Supplementary material for: Novel protective role for MAP kinase phosphatase 2 in inflammatory arthritis
Source: RMD Open. 2019 Jan 11;5(1):e000711. doi: 10.1136/rmdopen-2018-000711 (PMC6340532; doi:10.1136/rmdopen-2018-000711)
Supplement: Supplementary data [file rmdopen-2018-000711supp001.pdf]

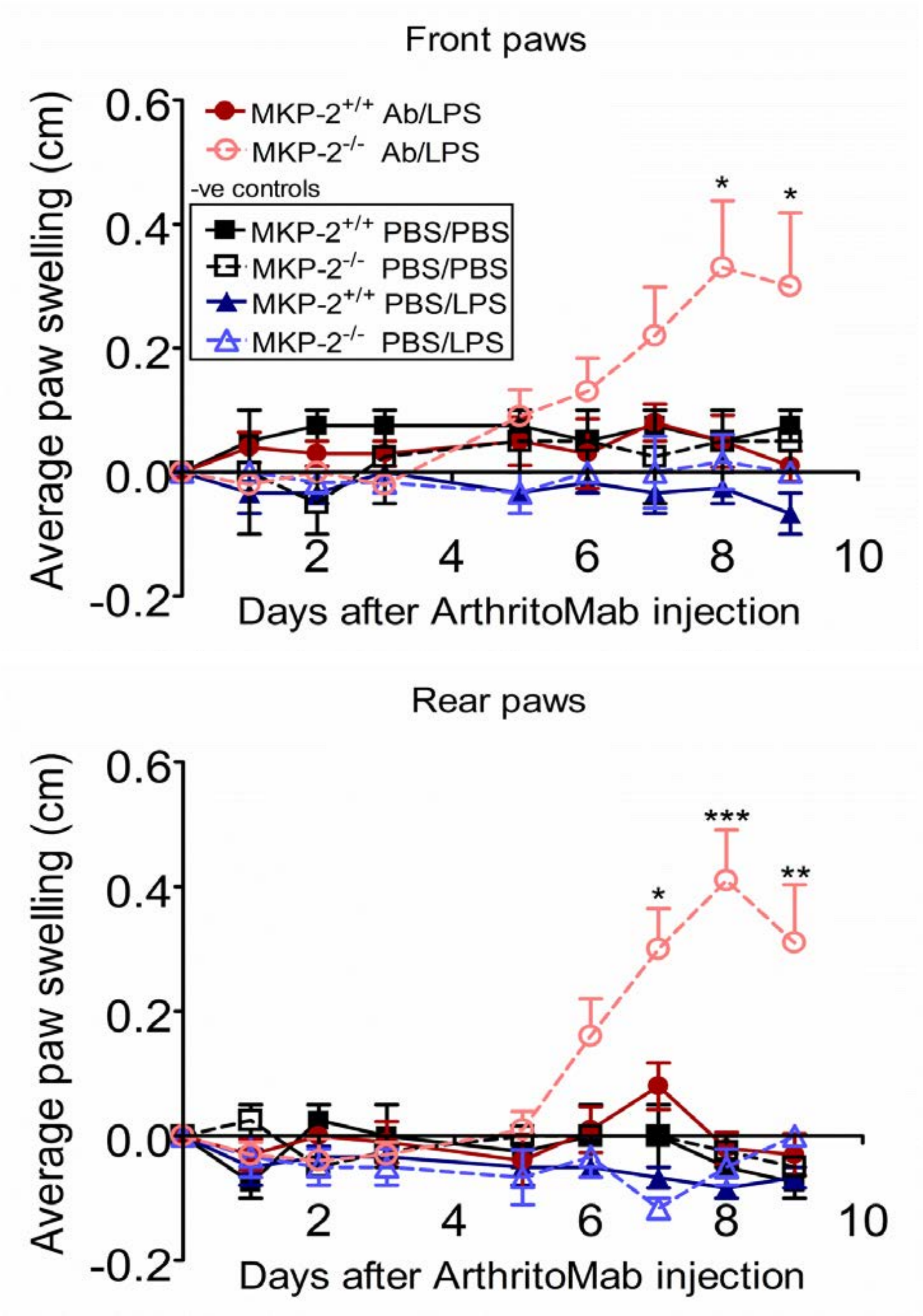

Fig. S1: Increased paw swelling in MKP-2 deficient mice in response to CAIA treatment.

Mice received a single dose of ArthritoMab at day 0 and LPS at day 3 (5 mice/genotype). Negative control groups received either PBS on both days (2 mice/genotype) or PBS on day 0 followed by LPS at day 3 (3 mice/genotype). Disease development was observed over a period of 9 days. Paw size was determined using a dial-gauge micrometer and averaged for front and back paws, respectively (MKP-$2^{+/+}$ (solid line/symbols), MKP-$2^{-/-}$ (dashed line/open symbols)). Error bars shown as standard error of the mean (SEM). * P≤0.05, ** P≤0.01, *** P≤0.001, two-tailed unpaired t test. N=3.
