# Supplementary material for: Novel protective role for MAP kinase phosphatase 2 in inflammatory arthritis
Source: RMD Open. 2019 Jan 11;5(1):e000711. doi: 10.1136/rmdopen-2018-000711 (PMC6340532; doi:10.1136/rmdopen-2018-000711)
Supplement: Supplementary data [file rmdopen-2018-000711supp002.pdf]

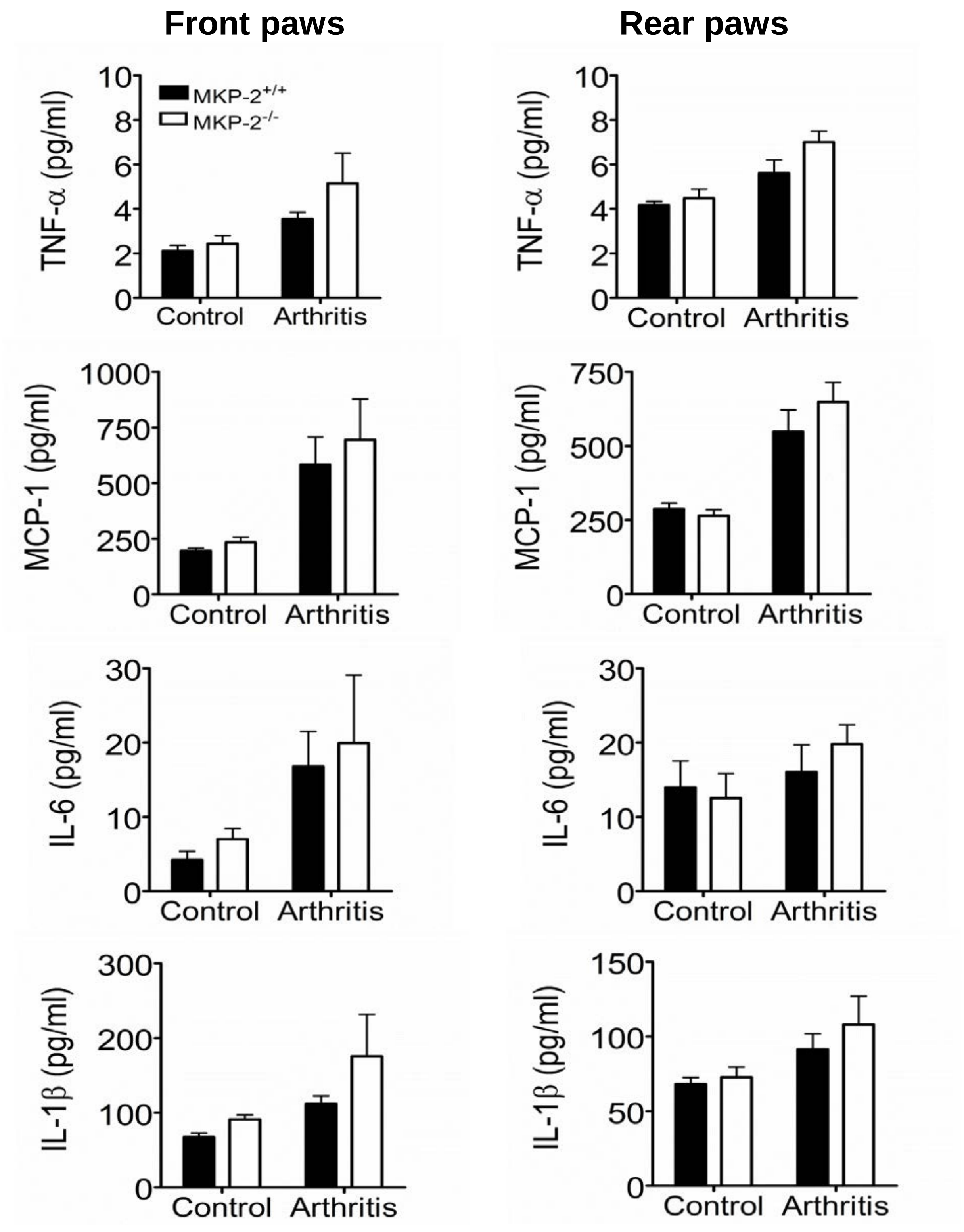

Fig. S2: Cytokine/chemokine concentration in the joints of MKP-2 deficient mice.
Front and rear paw joints from MKP-$2^{+/+}$ and MKP-$2^{-/-}$ mice (5 mice per group) were homogenized and extracts were tested for the presence of IL-6, IL-1β, TNF-α and MCP-1 using ELISA. Error bars are shown as standard error of the mean (SEM), two-tailed t-test, no significance. N=3
