# Supplementary material for: Novel protective role for MAP kinase phosphatase 2 in inflammatory arthritis
Source: RMD Open. 2019 Jan 11;5(1):e000711. doi: 10.1136/rmdopen-2018-000711 (PMC6340532; doi:10.1136/rmdopen-2018-000711)
Supplement: Supplementary data [file rmdopen-2018-000711supp004.pdf]

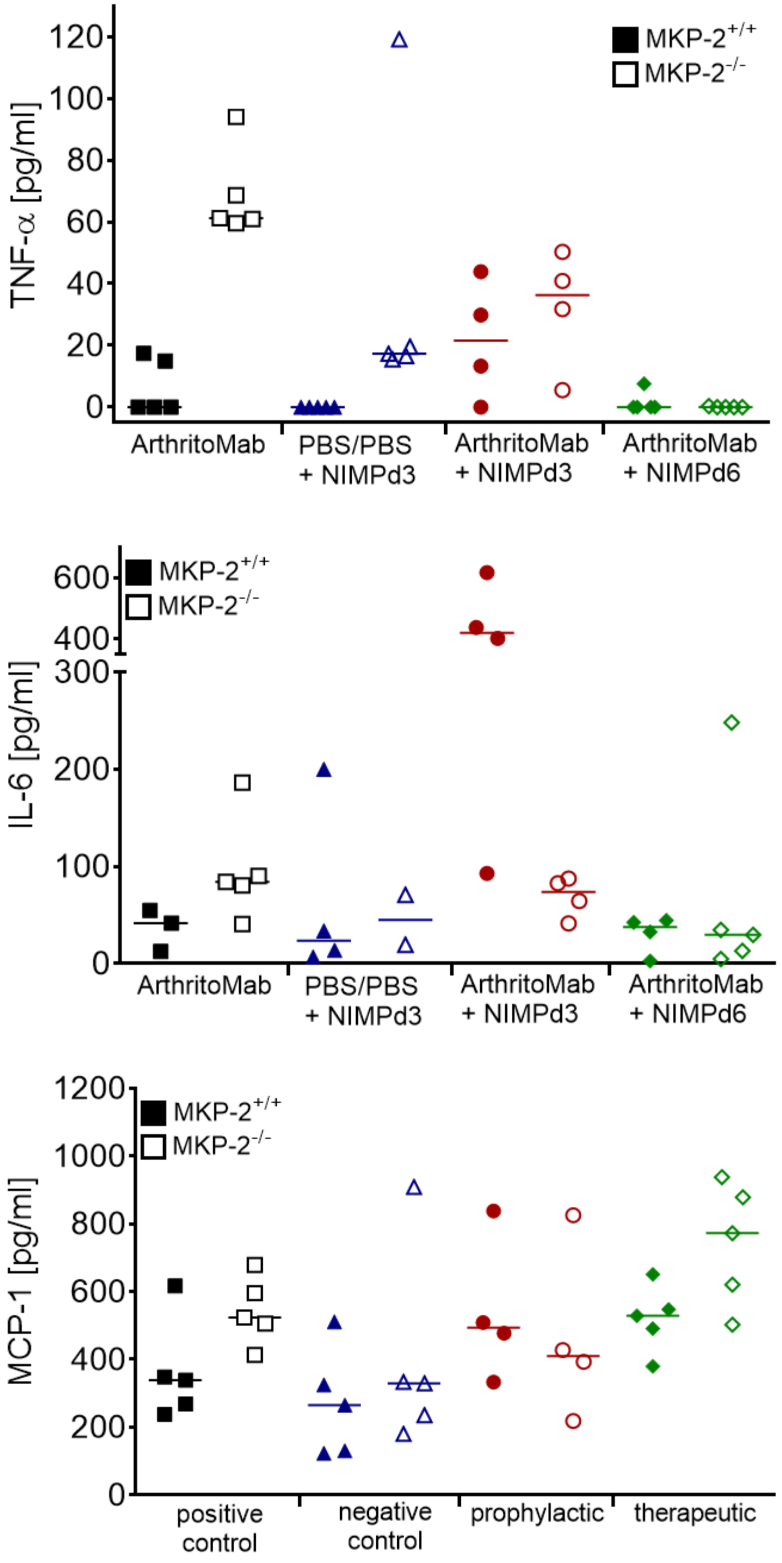

Fig. S4: Serum cytokines evaluated in control and neutrophil depletion mice

Serum from control groups, prophylactic and therapeutic groups (10 mice per group, 5 from MKP2$^{-/-}$ and 5 mice from MKP$^{+/+}$) were analysed for levels of TNFα, Il-6 and MCP-1, averaged and plotted as MKP-2$^{+/+}$ (solid bars) and MKP-2$^{-/-}$ (open bars). All error bars are shown as standard error of the mean (SEM). Representative of N=3
