# Supplementary material for: Novel protective role for MAP kinase phosphatase 2 in inflammatory arthritis
Source: RMD Open. 2019 Jan 11;5(1):e000711. doi: 10.1136/rmdopen-2018-000711 (PMC6340532; doi:10.1136/rmdopen-2018-000711)
Supplement: Supplementary data [file rmdopen-2018-000711supp003.pdf]

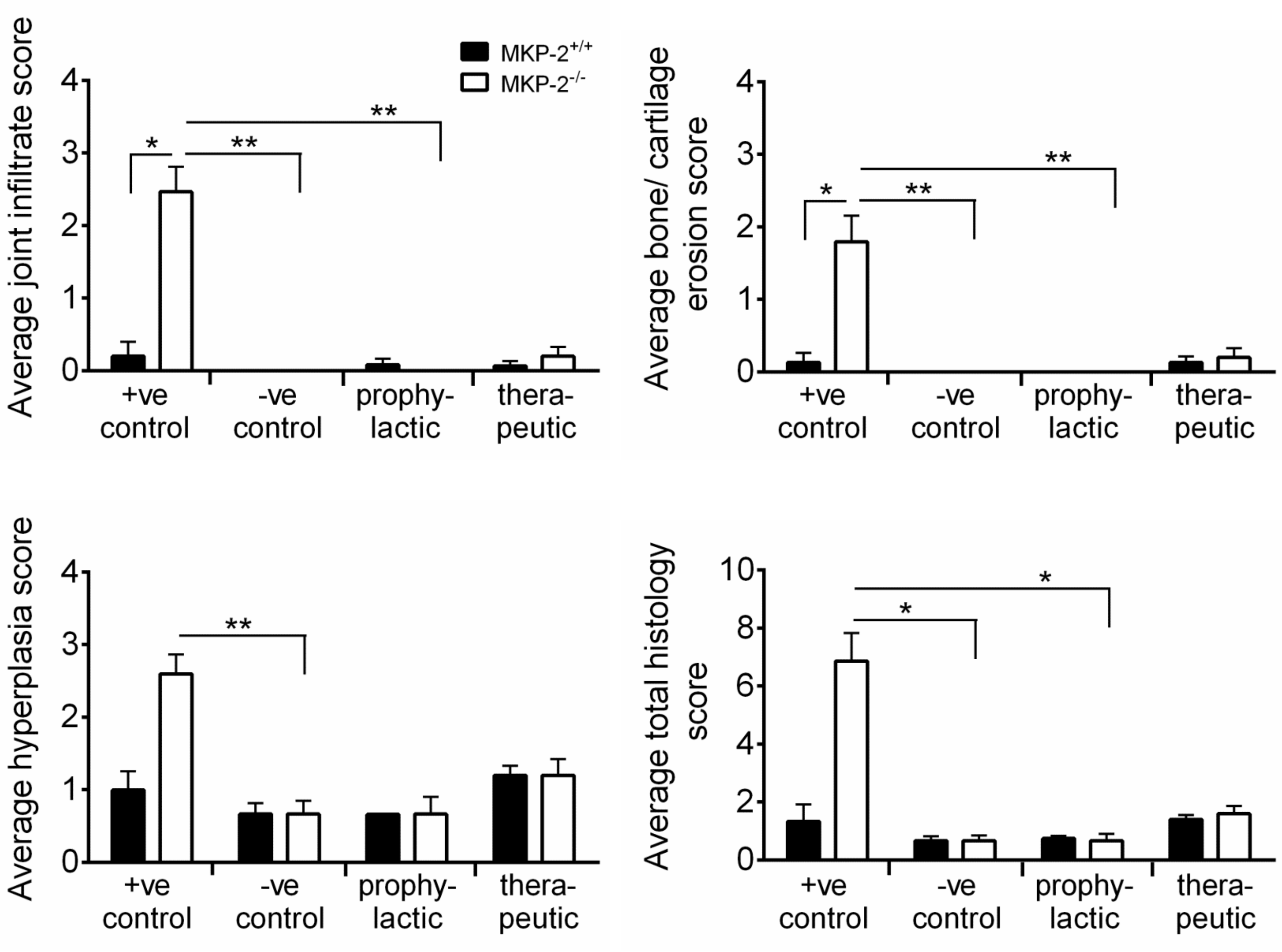

Fig. S3: Histological evaluation of the effects of prophylactic and therapeutic neutrophil depletion.

H&E stained tissue sections of front paw, rear paw and knee joints were scored blindly and evaluated for infiltration, hyperplasia and joint and bone erosion. Scores of all three joints were averaged per mouse and plotted as MKP-$2^{+/+}$ (solid bars) and MKP-$2^{-/-}$ (open bars). All error bars are shown as standard error of the mean (SEM). *$P<0.05$, **$P<0.01$, Kruskal-Wallis test with Dunn's post test. N=2
